# Patients in high- and low-revision hospitals have similar outcomes after primary knee arthroplasty: 1-year postoperative results from the Danish prospective multicenter cohort study, SPARK

**DOI:** 10.1007/s00167-023-07390-3

**Published:** 2023-04-12

**Authors:** Anne Mørup-Petersen, Michael Rindom Krogsgaard, Mogens Laursen, Frank Madsen, Matilde Winther-Jensen, Anders Odgaard

**Affiliations:** 1 Department of Orthopaedic Surgery, Copenhagen University Hospital Herlev and Gentofte, Gentofte Hospitalsvej 1, 2900 Hellerup, Denmark; 2 Department of Orthopaedic Surgery, Section for Sports Traumatology, Bispebjerg and Frederiksberg Hospital, University of Copenhagen, Bispebjerg Bakke 23, 2400 Copenhagen, NV Denmark; 3 Department of Orthopaedic Surgery, Aalborg University Hospital, Hobrovej 18-22, 9000 Aalborg, Denmark; 4 Department of Orthopaedic Surgery, Aarhus University Hospital, Palle Juul-Jensens, Boulevard 99, 8200 Aarhus N, Denmark; 5 Center for Clinical Research and Prevention, Department of Data, Biostatistics and Pharmacoepidemiology, Bispebjerg and Frederiksberg Hospital, Copenhagen, University of Copenhagen, Nordre Fasanvej 57, 2000 Frederiksberg, Denmark; 6 Department of Orthopaedic Surgery, Rigshospitalet, Copenhagen University Hospital, Blegdamsvej 9, 2100 Copenhagen, Denmark; 7 Department of Clinical Medicine, University of Copenhagen, Copenhagen, Denmark

**Keywords:** Knee arthroplasty, Knee replacement, Epidemiology, Patient-reported outcome measures, Revision rate variation, Regional variation, Knee range of motion, Radiographic classification, Patient selection, Osteoarthritis

## Abstract

**Purpose:**

It is well-known that revision rates after primary knee arthroplasty vary widely. However, it is uncertain whether hospital revision rates are reliable indicators of general surgical quality as defined by patients. The SPARK study compared primary knee arthroplasty surgery at three high-volume hospitals whose revision rates differed for unknown reasons.

**Methods:**

This prospective observational study included primary knee arthroplasty patients (total, medial/lateral unicompartmental and patellofemoral) in two low-revision hospitals (Aarhus University Hospital and Aalborg University Hospital Farsø) and one high-revision hospital (Copenhagen University Hospital Herlev-Gentofte). Patients were followed from preoperatively (2016–17) to 1-year postoperatively with patient-reported outcome measures including Oxford Knee Score (OKS), EQ-5D-5L and Copenhagen Knee ROM (range of motion) Scale. The surgical outcomes were compared across hospitals for patients with comparable grades of radiographic knee osteoarthritis and preoperative OKS. Statistical comparisons (parametric and non-parametric) included all three hospitals.

**Results:**

97% of the 1452 patients who provided baseline data (89% of those included and 56% of those operated) responded postoperatively (90% at 1 year)*.* Hospitals’ utilization of unicompartmental knee arthroplasties differed (Aarhus 49%, Aalborg 14%, and Copenhagen 22%, *p* < 0.001). 28 patients had revision surgery during the first year (hospital independent, *p* = 0.1) and were subsequently excluded. 1-year OKS (39 ± 7) was independent of hospital (*p* = 0.1), even when adjusted for age, sex, Body Mass Index, baseline OKS and osteoarthritis grading. 15% of patients improved less than Minimal Important Change (8 OKS) (Aarhus 19%, Aalborg 13% and Copenhagen 14%, *p* = 0.051 unadjusted). Patients with comparable preoperative OKS or osteoarthritis grading had similar 1-year results across hospitals (OKS and willingness to repeat surgery, *p* ≥ 0.087) except for the 64 patients with Kellgren–Lawrence grade-4 (Aarhus 4–6 OKS points lower). 86% of patients were satisfied, and 92% were “willing to repeat surgery”, independent of hospital (*p* ≥ 0.1). Hospital revision rates differences diminished during the study period.

**Conclusions:**

Patients in hospitals with a history of differing revision rates had comparable patient-reported outcomes 1 year after primary knee arthroplasty, supporting that surgical quality should not be evaluated by revision rates alone. Future studies should explore if revision rate variations may depend as much on revision thresholds and indications as on outcomes of primary surgery.

**Level of evidence:**

Level II (Prospective cohort study).

## Introduction

When hospitals differ in cumulative revision rates (CRR) following primary knee arthroplasty (KA), it leads to assumptions of differences in the quality of surgery. This is the case for variation both among and within countries [22]. However, with pain relief and regaining knee function being the primary goals of KA surgery, CRRs may not be the most important measure of treatment quality in the vast majority of patients, i.e., those, who are never revised and whose spectrum of postoperative results are not reflected in the statistics [8]. In 2015, Danish KA surgeons recognized the need for a comprehensive comparison of the primary KA surgery performed in the three (of five) administrative regions with the greatest differences in 1-, 2-, 5- and 10-year CRRs. The goal was to determine whether CRR variations were a reflection of overall differences in the quality of primary KA surgery, defined as patients’ subjective improvement after surgery [26]. By posing this question, the focus was shifted from registry-based quality to quality based on patient assessment, patient-reported outcome measures (PROMs) and range of motion.

Each region was represented by its largest KA hospital in a prospective observational cohort study, SPARK (“Variation in patient Satisfaction, Patient-reported outcome measures, radiographic signs of Arthritis, and Revision rates in Knee arthroplasty patients in three Danish regions”). The baseline publication [16] from the SPARK study compared the three hospitals’ patient selection at primary surgery by analyzing preoperative data from 1452 patients who underwent primary KA in routine clinical settings. It was reported that patient demographics, anxiety and depression symptoms, KA incidence, implant selection and radiographic classification of knee osteoarthritis varied somewhat between hospitals, but preoperative PROMs did not. The majority of hospital differences at baseline were in opposition to well-known revision risk factors. For example, one low-revision hospital (Aarhus) used more unicompartmental implants (49%) than the others [Aalborg 14% (low-CRR) and Copenhagen 22% (high-CRR)] (Table 1). Overall, the study was unable to identify differences in baseline characteristics that could adequately explain the persistent hospital differences in CRRs [16]. The present follow-up study compares the postoperative outcomes of primary KA in the SPARK cohort and determines if patients with a certain level of symptoms or radiological knee osteoarthritis (OA) can expect similar outcomes at the three hospitals.

**Table 1:** Baseline characteristics of participants

	Value/count	%
Patients (*n*) (male)	1452 (659)	100 (45)
Age (years) (mean [median] ± SD)	68.0 [69] ± 9	
Body Mass Index (kg/m^2^) (mean [median] ± SD)	29 [28] ± 5	
Patients per hospital, all implants (UKA^a^)	1452 (393)^a^	100 (27)
Aarhus University Hospital (low-revision)	321 (157)	22^b^ (49)^c^
Aalborg University Hospital Farsø (low-revision)	202 (28)	14^b^ (14)^c^
Copenhagen University Hospital Herlev–Gentofte (high-revision)	929 (208)	64^b^ (22)^c^
Radiographic severity of knee osteoarthritis (*n* total)	1051	(100)
Kellgren–Lawrence classification		
≥ 2 (*n*)	987	94
≥ 3 (*n*)	851	81
Ahlbäck classification		
≥ 2 (*n*)	704	67
≥ 3 (*n*)	305	29

Patient characteristics. For complete baseline results per hospital, we refer to the baseline publication

^a^UKA here denote the proportion of patients who had a unicompartmental implant inserted (medial, lateral or patellofemoral)

^b^Hospital proportion of SPARK sample. ^c^UKA proportion of local hospital sample

Aside from differences in CRR between regions (e.g., 1.0, 2.2 and 5% per 2 years in 2015) and undocumented claims of cultural differences between east (Capital, high-revision) and west (low-revision), no previous data had led to hypotheses of quality differences across the country. Therefore, all data were analyzed under the assumption (null hypothesis) that there was no difference in hospital outcomes.

## Materials and methods

The study was ethically approved by The National Committee of Health Research Ethics (Protocol no. 16038343, 2 September 2016). All patients provided written consent to participate in the study. The study was reported in accordance with the STROBE guidelines (STrengthening the Reporting of OBservational studies in Epidemiology). Register data were retrieved from the Danish Knee Arthroplasty Register and the Danish National Patient Register [24].

### Patient inclusion

The prospective observational cohort study, SPARK, was conducted in three high-volume hospitals with 2-year CRRs that were representative of their region during the preceding 3 years: Aarhus University Hospital (1.9%) in Central Denmark Region (2.5%), Aalborg University Hospital Farsø (1.6%) in North Denmark Region (1.5%) and Copenhagen University Hospital Herlev-Gentofte (5.6%) in the Capital Region (4.7%) [16]. From 1 Sep 2016 to 31 Dec 2017, surgeons and employed medical students invited patients scheduled for KA (total (TKA), medial or lateral unicompartmental (MUKA/LUKA), or patellofemoral (PFA) knee arthroplasty) to participate in the study. Knee tumors, severe developmental lower limb deformities, haemophilia, dementia and severe language barriers were exclusion criteria, and this follow-up study only enrolled the 1452 patients who had provided PROM-data before surgery [16]. Questionnaires were sent by emails with unique links before surgery and 6 weeks, and 3, 6 and 12 months postoperatively. Patients who could only respond via traditional mail were only allowed inclusion in the final 6 months of the inclusion period. Some patients participated with both knees at separate occasions, and many more had bilateral knee trouble, thus the emails specifically addressed “right” or “left knee” and current follow-up time. If necessary, two reminders were sent, and at 1 year, a printed questionnaire with a postage-paid envelope was sent to non-responders. Surgeons continuously delivered surgical information as part of their routines and any missing or erroneous information was meticulously corrected using medical records.

Participants who underwent revision during the first year were not the main focus of the study. However, their PROMs were collected and reported until the day of revision. For example, a patient who was revised after 8 months could contribute to PROM analyses at 6 months, but not at 1 year. All subsequent revisions were attributed to the primary KA hospital regardless of which hospital (public or private) performed the revision. Minor surgery, such as wound debridement or manipulation under anesthesia did not result in participation cessation.

### Radiographic classification of knee osteoarthritis

Blinded posteroanterior weight-bearing radiographs were used to evaluate the radiographic severity of knee osteoarthritis [23]. To facilitate fair comparisons, LUKA and PFA patients and radiographs with predominantly lateral joint space narrowing were excluded. Two radiologists graded knee OA according to Kellgren–Lawrence (K–L) classification (0–4, 4 most severe) and Ahlbäck score (0–5, 5 most severe) [16, 1, 12]. Moreover, 13 experienced KA surgeons performed thousands of “head-to-head” comparisons of the radiographs based on heuristics, i.e., “rules of thumb” and clinical experience and without the use of traditional classification systems, resulting in a complete OA severity ranking of all radiographs (further details in preceding publications [16, 18]).

### Patient-reported outcome measures (PROMs)

The primary outcome, Oxford Knee Score (OKS, 0–48, 48 best), was reported as absolute score, change from baseline, and as proportions of patients achieving the Minimal Important Change (MIC) of 8 points indicating an important improvement for the average patient at 1 year [2, 6, 10, 11, 17, 25]. Copenhagen Knee ROM Scale (CKRS) assessed patient-reported passive range of motion (ROM), i.e., flexion (0–6, 6 max) and extension (0–5, 5 max) as well as estimated proportions of patients with flexion or extension deficits [14]. Every PROM set began with the generic EQ-5D-5L and EQ-VAS and a “global knee anchor” question asking, “How is your knee?” (VAS, 0–100, 100 best), and patients reported how often they used any type of analgesics for knee pain [30].

Additional questions and PROMs were added at varying time points. At baseline, height, weight, smoking, alcohol and level of urbanization data were reported [13, 29]. The Forgotten Joint Score (FJS) [3] and UCLA Activity Scale (UCLA) [15] were added from 3 months postoperatively, and UCLA was also used at baseline. At 6 months, patients answered whether they had received physiotherapeutic assistance in rehabilitation after hospital discharge. From 3 months on, patient satisfaction was measured by asking, “How satisfied are you with the overall experience of the operation and its result?” (five Likert boxes, one neutral). As the answers could be influenced by experiences related to hospital service, kindness of caretakers, etc. [5], also “willingness to repeat surgery” was reported at 1 year: “Suppose you could turn back time, would you still choose to have a knee replacement now that you know the outcome?” (five Likert boxes, one neutral).

### Implants, perioperative care, and follow-up routines

The SPARK study did not interfere with local hospital routines concerning, e.g., analgesics use, aftercare, or selection of KA implants. Each hospital used a unique selection of cemented, uncemented and hybrid implants that were on the market for at least 10 years and had proven good survival in registries [24]. The predominant systems were NexGen^™^ (Zimmer Biomet), PFC^™^ Sigma (DePuy Synthes), Triathlon^™^ (Stryker), Oxford^™^ Mobile Bearing and ZUK^™^ (Zimmer Biomet) and Avon^™^ (Stryker).

In all three hospitals, tranexamic acid, glucocorticoids, local anesthetics and prophylactic antibiotics (dicloxacillin in Copenhagen, cefuroxime in Aarhus and Aalborg) were administered intraoperatively. Paracetamol, non-steroid anti-inflammatory drugs (NSAID) and opioids were the oral analgesics of choice for up to 4 weeks postoperatively. In 2017, the average length of stay for TKA patients in Aarhus, Aalborg and Copenhagen was 2.4, 1.4 and 2.2 nights, respectively, and for MUKA patients it was 0.6, 1.3 and 0.7 nights [27].

The routine preoperative multidisciplinary patient seminar included preparation training with physiotherapists (crutch walking, stair climbing, etc.). Postoperatively, Aalborg and Copenhagen patients were trained by a physiotherapist, and Copenhagen patients were routinely offered free of charge supervised physiotherapy upon discharge. Aarhus and Aalborg patients were screened 2–6 weeks after discharge to identify those in need of physiotherapy and only those whose progress was unsatisfactory after 6–8 weeks or who had abnormal findings on 1-year radiographs were referred to the surgeon for a follow-up appointment. In contrast, all Copenhagen patients saw their surgeon and had radiographs taken after 3 months.

### Statistics

Based on pragmatic considerations and feasibility, the study aimed for a sample size of 1080 patients (75% inclusion rate and 80% response rate among 1800 patients) [16]. All significance tests comprised all three hospitals unless otherwise specified. In regression analyses, Aarhus was selected as the reference hospital, because it was situated between the other two hospitals in terms of geography, urbanization level, and revision rates. All observations were treated as independent data [21]. *P* values were two-sided with alpha level 0.05. Standard deviations were displayed as “(± SD)”. Tabular data were analyzed by Chi-square test (with Monte-Carlo correction for expected cell counts < 5), and Clopper–Pearson confidence intervals (95% CI) were provided when relevant. Non-parametric (ranked) methods (Kruskal–Wallis or Wilcoxon/Mann–Whitney *U* test) were used for ordinal measures (UCLA, global knee anchor, patient satisfaction, willingness to repeat, use of analgesics and radiographic classifications, while parametric methods (one-way analysis of variance (ANOVA) or *t* test) were applied to OKS, FJS, EQ-5D and CKRS [17]. Multiple linear regression analyses (dependent variable OKS) were conducted with both Ahlbäck and K–L, but since the overall result was not changed with classification method, only Ahlbäck-based confidence intervals (CIs) were reported. Since 1-year change scores for revised patients were unavailable, the analysis of change in OKS was repeated using imputed results (clearly specified). Analyses were carried out in R (RStudio) in Mar 2019 [20]. Data collection and Case Report Forms (CRF) were handled by Procordo Software Aps, Copenhagen.

## Results

### Patient inclusion

Baseline data were available for 1452 patients (68.0 ± 9 years, 45% males, 89% response rate) (Table 1). The 41 patients who participated by mail were 8 years older and more likely to be female (71%) than those who participated online. According to the post-hoc inclusion analysis, 56% of patients (62% in Aarhus and Copenhagen, 38% in Aalborg) provided baseline data for the SPARK study in 2017 [16]. Participants were younger than non-participants (67.7 ± 9 vs. 68.8 ± 11 years, *p* = 0.02) and more likely to be male (42 vs. 38%, *p* = 0.016). Implant types were equally distributed among participants and non-participants within each hospital (*p* ≥ 0.2) [16].

1414 patients (97%) responded postoperatively at least once, and 1307 (90%) responded at 1 year (Table 2). The response rate was comparable among hospitals (*p* = 0.4). In the first year, three patients left the study, seven died, and nine were lost to follow up due to errors, such as incorrect laterality or change of email address. Revision surgery was performed on 28 patients (1.9%) during the first postoperative year; 2 (0.6%) in Aarhus, 4 (2.0%) in Aalborg and 22 (2.4%) in Copenhagen (*p* = 0.1). Last available postoperative OKS, revision time and indication were listed for each patient (Table 3). Deep infection caused 13 revisions (1 (0.3%) in Aarhus, 1 (0.5%) in Aalborg and 11 (1.2%) in Copenhagen, *p* = 0.4).

**Table 2:** Postoperative response rates

	Baseline	6 weeks	3 months	6 months	1 year	Any postop
Responding patients (*n*)	1452	1147^a^	1237	1241	1307	1414
Available patients (*n*)	1452	1296^a^	1435	1433	1417	1443
Revised/dead patients (*n*)	0/0	9/1	15/2	17/2	28/7	9/1
Response rate						
Per available patients (%)	(100)^a^	89	86	87	92	98
Per 1452 baseline responders (%)	100^a^	79	89	86	90	97
Days from surgery (mean [median] ± SD)	− 29 [− 18] ± 32	39 [38] ± 7	87 [84] ± 14	179 [176] ± 14	368 [359] ± 27	–

^a^The 6-week questionnaire was delayed and thus not sent to the first 146 included patients

**Table 3:** Characteristics of patients who were revised during the first postoperative year

Patient group	*N* (%)	Male sex (*n* (%))	Age years (mean ± SD)	Body mass index kg/m^2^ (mean ± SD)	Implant type (TKA/MUKA/other)
No revision	1424 (98)	642 (45)	68.0 ± 9	28.9 ± 5	1039/328/57
Revision	28 (2)	17 (61)	66.4 ± 10	26.9 ± 4 (CI -0.6-(-3))	20/8/0
*p*	–	0.1	0.4	0.008	0.7

Revision time		Aarhus *n* = 321	Aalborg *n* = 202	Copenhagen *n* = 929	Total sample *n* = 1452
0–6 w	*n*	1	1	7	9
	Indication	A	A	A, A, A, A, A, B, B	7A, 2B
	Last OKS before revision	–	–	–	–
6 w.3 mo	n	0	0	6	6
	Indication			A, A, A, A, B, C	4A, 1B, 1C
	Last OKS before revision			A: 34,39,*NA*,*NA*. B: 25. C: 28	Mean: (32)
3–6 mo	*n*	1	1	0	2
	Indication	C	C		2C
	Last OKS before revision	10	20		Mean: 15
6–12 mo	*n*	0	2	9	11
	Indication		C, C	A, A, B, C, C, C, C, C, C	2A, 1B, 8C
	Last OKS before revision		11,35	A: 45,18. B: 26. C: 16,28,29,31,32,34	Mean: 28
Total	Revised (n) during year 1	2	4	22	28
	Indications	1A, 1C	1A, 3C	11A, 4B, 7C	13A, 4B, 11C
	Revision rate in sample (%)	0.6	2.0	2.4	1.9
	95% CI (%)	0.7–2	0.5–5	2–4	1–3

Indications: Revision due to *A* deep infection, *B* fracture or liner dislocation, *C* other cause. *“Last OKS before revision”* Patient’s last postoperative Oxford Knee Score before revision. *NA* Missing (not available)

### Radiographic classification of knee osteoarthritis

Blinded K–L and Ahlbäck classifications of OA severity were made for 1051 radiographs (86% available of 1228, after exclusions) and radiographs were ranked from no. 1 to 1051 (no. 1 most severe) based on surgeons’ 17,767 direct comparisons [16, 18].

### Patient-reported outcome measures (PROMs)

**Fig. 1:**
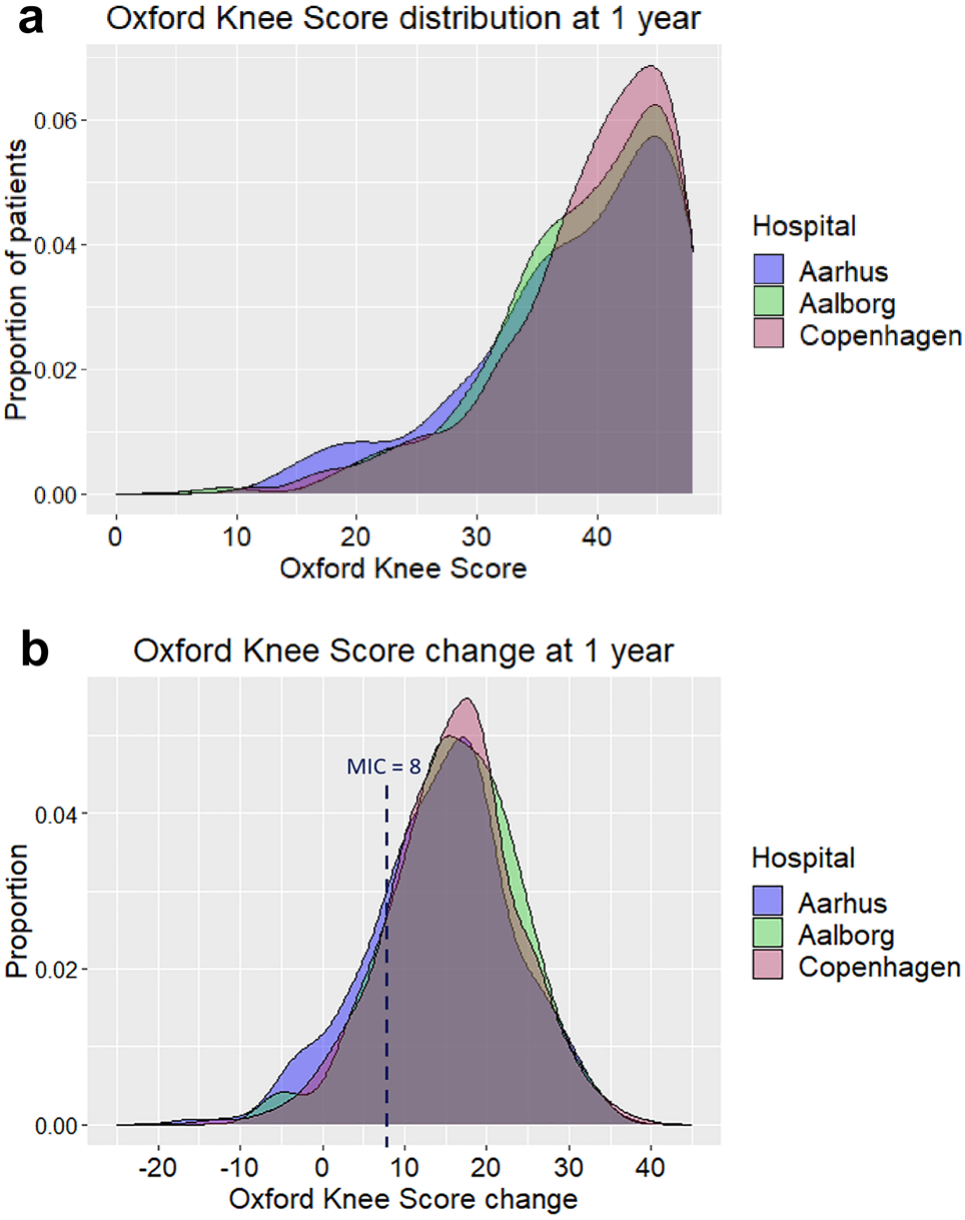
**a**, **b** Oxford Knee Score at 1 year. **A** Absolute score and **B** change score with minimal important change (MIC) = 8 points

OKS at 1 year did not differ significantly among the three hospitals (39 ± 7, p = 0.1) (Fig. 1a, Table 4), nor when adjusted for age and sex, or when further adjusted for baseline OKS and EQ-VAS and variables that differed among hospitals preoperatively, i.e., BMI, anxiety and depression symptoms, and radiographic classification (Ahlbäck or K–L). OKS change at 1 year was lower in Aarhus (+ 1.6 in Aalborg, CI 0.07–3, and + 1.3 in Copenhagen, CI 0.2–2, respectively) (Fig. 1b, Table 4). This conclusion was partially modified by adjusting for age, sex and baseline OKS (+ 1.0 in Aalborg, CI -0.3–2, + 1.1 in Copenhagen, CI 0.2–2), and when additional adjustments were made for BMI, EQ-VAS, anxiety and depression and radiographic classification, there were no significant differences between hospitals (Aalborg CI -0.4–3, Copenhagen CI -0.1–2, *p* > 0.2).

**Table 4:** Patient-reported outcomes at 1-year follow-up

	Total sample	Aarhus (low rev. rate)	Aalborg (low rev. rate)	Copenhagen (high rev. rate)	*P*
Included at baseline (*n*) (male %)	1452 (45)	321 (45)	202 (56)	929 (43)	0.002
Implant type, *n* (%)					< 0.001
TKA	1059 (73)	164 (51)	174 (86)	721 (78)	
MUKA	336 (23)	129 (40)	25 (12)	182 (20)	
PFA	50 (3)	23 (7.2)	3 (1.5)	24 (2.6)	
LUKA	7 (1)	5 (1.6)	0 (0.0)	2 (0.2)	
Preoperative radiographic knee osteoarthritis					
Kellgren–Lawrence classification ≥ 2 (%)	987 (94)	202 (98)	156 (91)	629 (93)	0.01
Ahlbäck score ≥ 2 (%)	704 (67)	154 (75)	106 (62)	444 (66)	0.02
Surgeons’ ranking (mean [IQR 25–75%])	540 [270–808]	380 [188–718]	598 [315–864]	561 [293–824]	< 0.001
Oxford knee score (OKS)					
Preoperative	23.3 [24] ± 7	23.5 [24] ± 7.0	23.2 [24] ± 6.5	23.3 [24] ± 6.7	0.9
1 y. (*n* = 1307)	39 [41] ± 7	38.1 [40] ± 8.3	39.1 [41] ± 7.2	39.2 [41] ± 7.2	0.09
Last available postop. (*n* = 1414)	38 [40] ± 8	37.5 [40] ± 8.7	38.7 [40] ± 7.5	38.5 [40] ± 7.8	0.1
Change 1.y (*n* = 1307)	15 ± 8	14.3 ± 8.7	15.9 ± 7.8	15.7 ± 8.0	0.04
OKS change < MIC (8 points), *n* (%)					
1 y. (*n* = 1307)	195 (15)	56 (19)	25 (13)	114 (14)	0.051
1 y. imputed^a^ (*n* = 1335)	223 (17)	58 (20)	29 (15)	136 (16)	0.2
Last available postop. (*n* = 1414)	237 (17)	66 (21)	31 (16)	140 (16)	0.07
Willingness to repeat surgery (%)					0.1
“Yes, certainly”	1005 (77)	211 (73)	150 (80)	644 (77)	
“Yes, probably”	200 (15)	46 (16)	26 (14)	128 (15)	
“I don’t know”	52 (4)	14 (4.9)	6 (3)	32 (3.9)	
“No, probably not”	32 (2.5)	12 (4.2)	3 (1.6)	17 (2.0)	
“No, absolutely not”	17 (1.3)	5 (1.7)	2 (1.1)	10 (1.2)	
Patient satisfaction (%)					
“Satisfied” or “very satisfied”	1125 (86)	238 (83)	161 (87)	726 (87)	0.6^b^
Global knee anchor (0–100)					
Preoperative	28 ± 18	27 ± 17	30 ± 18	29 ± 18	0.2
1 y	80 ± 21	78 ± 24	81 ± 21	80 ± 19	0.08
Change	51 ± 26	50 ± 29	51 ± 26	51 ± 25	0.8
Forgotten joint score					
1y	60 ± 27	59.1 ± 29	59.7 ± 25	60.1 ± 26	0.9
Knee range of motion (CKRS units)					
Flexion					
Preoperative	4.9 [5]± 1.2	4.8 [5] ± 1.2	4.8 [5] ± 1.1	4.9 [5] ± 1.2	0.2
1y	5.4 [6] ± 0.8	5.41 [6] ± 0.76	5.30 [5] ± 0.76	5.34 [5]± 0.77	0.3
Deficit (CKRS 0–4) (n (%))	165 (13)	32 (11)	21 (11)	112 (13)	0.5
Change	0.48 [0] ± 1	0.57 [0] ± 1.2	0.55 [0] ± 1.2	0.43 [0] ± 1.1	0.2
Extension					
Preoperative	3.5 [4] ± 1.0	3.4 [4] ± 1.0	3.4 [3] ± 0.9	3.5 [4] ± 0.9	0.2
1 y	4.1 [4] ± 0.7	4.24 [4] ± 0.65	4.10 [4] ± 0.61	4.12 [4] ± 0.68	0.02
Deficit (CKRS 0–3) (*n* (%))	161 (12)	29 (10)	24 (13)	108 (13)	0.4
Change ^c^	0.7 [1] ± 1	0.73 [1] ± 1.0	0.72 [1] ± 0.9	0.64 [1] ± 1.0	0.6
UCLA activity scale					
Preoperative	4.7 [4] ± 2	4.8 [4] ± 1.9	4.8 [4] ± 1.9	4.7 [4] ± 1.8	0.6
1 y	6.0 [6] ± 2	5.8 [6] ± 1.9	6.0 [6] ± 1.8	6.0 [6] ± 1.9	0.5
Change	1.2 [1] ± 2	1.0 [1] ± 1.9	1.3 [1] ± 1.9	1.3 [1] ± 1.9	0.06
EQ-VAS					
Preoperative	61 ± 22	62 ± 21	58 ± 24	62 ± 22	0.1
1 y	79 ± 18	78 ± 20	82 ± 15	79 ± 18	0.08
Change	17 ± 23	16.1 ± 24	24.3 ± 24	16.3 ± 22	< 0.001
EQ-5D-5L index					
Preoperative	0.59 ± 0.2	0.59 ± 0.15	0.61 ± 0.12	0.59 ± 0.15	0.1
1 y	0.81 ± 0.2	0.80 ± 0.17	0.83 ± 0.14	0.82 ± 0.14	0.04
Change	0.22 ± 0.2	0.20 ± 0.18	0.23 ± 0.15	0.22 ± 0.17	0.049
Daily use of analgesics for knee pain, 1 y. (*n* (%))	166 (13)	41 (14)	22 (12)	103 (12)	0.4^a^
Supervised physiotherapy in rehabilitation, 1 y. (*n* (%)) ^d^	702 (73)	115 (51)	92 (70)	495 (81)	< 0.001

When no unit was noted, means ± SD [and medians] were provided. ^a^1y. imputed”: The 28 revised patients were here assumed to be in the group with OKS change < MIC (8 points). ^b^Patient satisfaction was dichotomized for presentation, but *P* value refers to tests of all 5 ordinal answer options. ^c^Only the last 699 patients were included in this analysis due to delay of scale development. ^d^Only the last 966 included patients were asked about physiotherapy in rehabilitation

Abbreviations: “Surgeons’ ranking” refers to radiographs ranked by 13 surgeons from 1 to 1051, where no. 1 has most severe osteoarthritis.* MIC* Minimal Important Clinical difference (8 points). *CKRS* Copenhagen Knee ROM Scale is patient-reported flexion from 0 (unable) to 6 (full flexion ability), and extension from 0 (unable) to 5 (full extension or slight hyperextension). In development studies, “flexion deficit” (< 5) identified 95% of patients with passive flexion below 100° (sensitivity) and excluded 81% of patients with flexion above 100° (specificity). “Extension deficit” (< 4) identified 78% of patients with passive extension worse than 10° (sensitivity) and excluded 70% of patients with extension better than 10° (specificity)

At 1 year, 19% of patients in Aarhus, 13% in Aalborg, and 14% in Copenhagen did not attain the MIC of 8 OKS points (*p* = 0.051). To fairly account for the distribution of revised patients at 1 year, a new analysis was conducted in which all 28 revision patients, that were excluded from the latter analysis, were now assigned an imputed (hypothetic) change score below MIC. The new proportions of patients not attaining MIC in the three hospitals were now 20%, 15% and 16%, respectively (*p* = 0.2). Comparing the “last available” postoperative OKS change score of 1414 patients with at least one postoperative response (including 17 revision patients), hospitals did not differ significantly (21, 16 and 16%, respectively, *p* = 0.07).

**Fig. 2:**
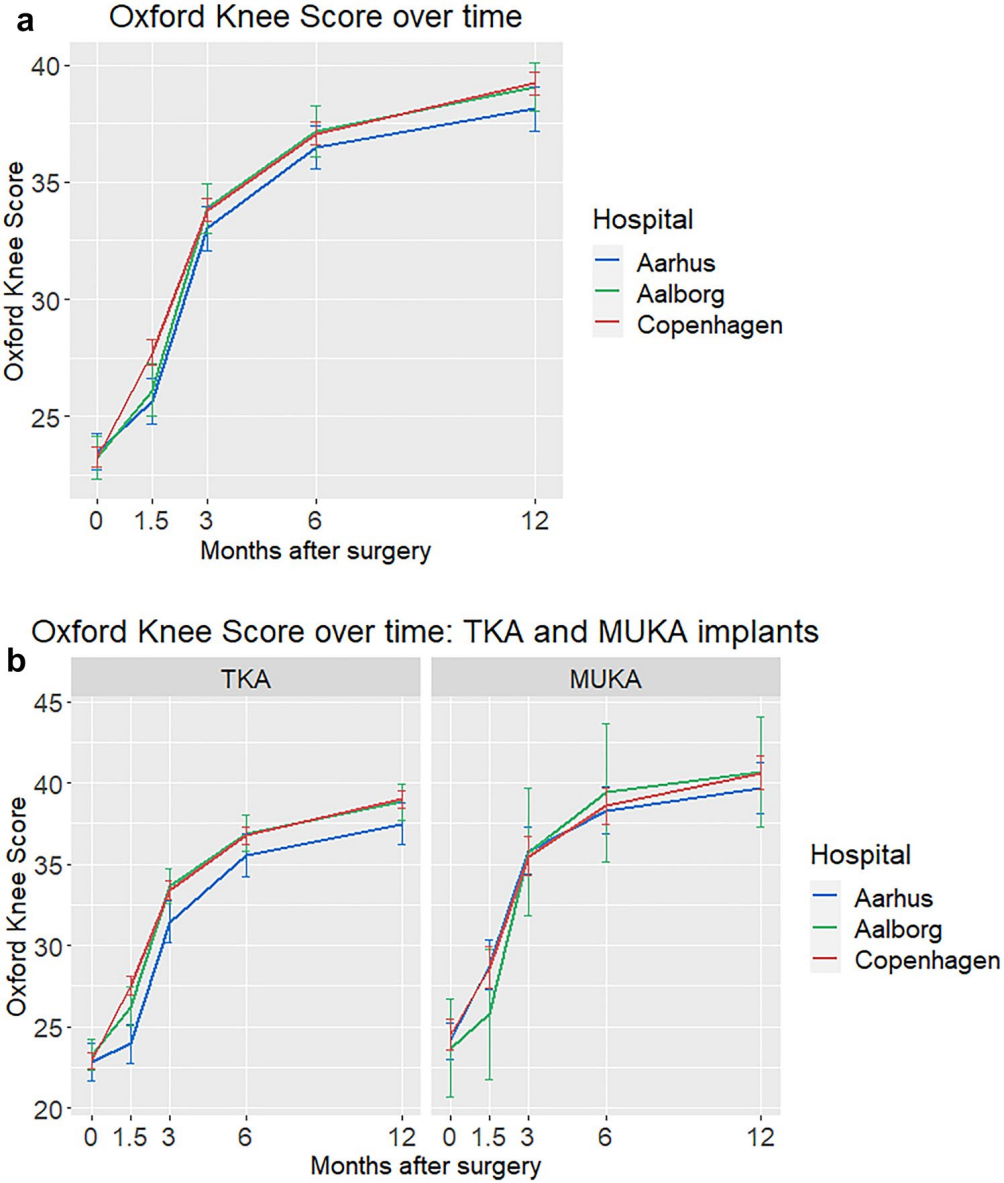
**a**, **b** Oxford Knee Score during the first postoperative year in **A** all patients, and in **B** TKA and MUKA patients separately. Whiskers denote mean ± 2 × std. error of the mean

When studied over time, OKS was higher at 6 weeks in Copenhagen (27.7 ± 7) than in Aarhus (25.6 ± 8) and Aalborg (26.1 ± 7) (*p* = 0.001, unadjusted) (Fig. 2a). This hospital difference was nuanced when MUKA and TKA patients were studied separately (Fig. 2b), and other PROMs did not vary over time between hospitals (Table 5 depicts the complete sample). Through the entire study period, OKS differed between TKA and MUKA patients, e.g., 1-year OKS was 38.7 and 40.3, respectively (CI 0.6–3).

**Table 5:** Development of main PROMs over time after surgery (all hospitals)

	Preoperative	6 weeks	3 months	6 months	1 year
Oxford knee score (OKS)	23 ± 7	27 ± 8	34 ± 8	37 ± 7	39 ± 7
OKS change	–	3.6 ± 8	10 ± 8	14 ± 8	15 ± 8
OKS change < MIC (8 points) (n (%))	–	788 (69)	462 (36)	262 (21)	195 (15)
Global knee anchor (0–100)	28 ± 18	60 ± 21	71 ± 22	76 ± 21	80 ± 21
Knee range of motion (CKRS units)					
Flexion	4.9 ± 1.2	4.5 ± 1.1	5.0 ± 0.9	5.3 ± 0.8	5.4 ± 0.8
Deficit (CKRS 0–4) (*n* (%))	416 (29)	525 (46)	317 (25)	188 (15)	165 (13)
Extension	3.5 ± 0.9	3.5 ± 0.7	3.9 ± 0.8	4.0 ± 0.7	4.1 ± 0.7
Deficit (CKRS 0–3) (*n* (%)) ^a^	340 (49)^a^	336 (42)^a^	246 (27)^a^	202 (17)	161 (12)
Forgotten Joint Score	–	–	43 ± 25	53 ± 26	60 ± 27
UCLA Activity Scale	4.7 [4] ± 2	–	5.4 [5] ± 2	5.8 [6] ± 2	6.0 [6] ± 2
Daily use of analgesics against knee pain (*n* (%))	854 (59)	870 (76)	498 (39)	274 (22)	166 (13)
EQ-5D VAS	61 ± 22	71 ± 18	76 ± 17	78 ± 18	79 ± 18
EQ-5D-5L Index	0.59 ± 0.2	0.70 ± 0.1	0.76 ± 0.1	0.79 ± 0.1	0.81 ± 0.2

When no unit was noted, means, ± SD and [medians] were provided

Abbreviation: *CKRS* Copenhagen Knee ROM (Range of motion) Scale

^a^In CRKS extension, total n was increasing during the study due to concomitant scale development

**Fig. 3:**
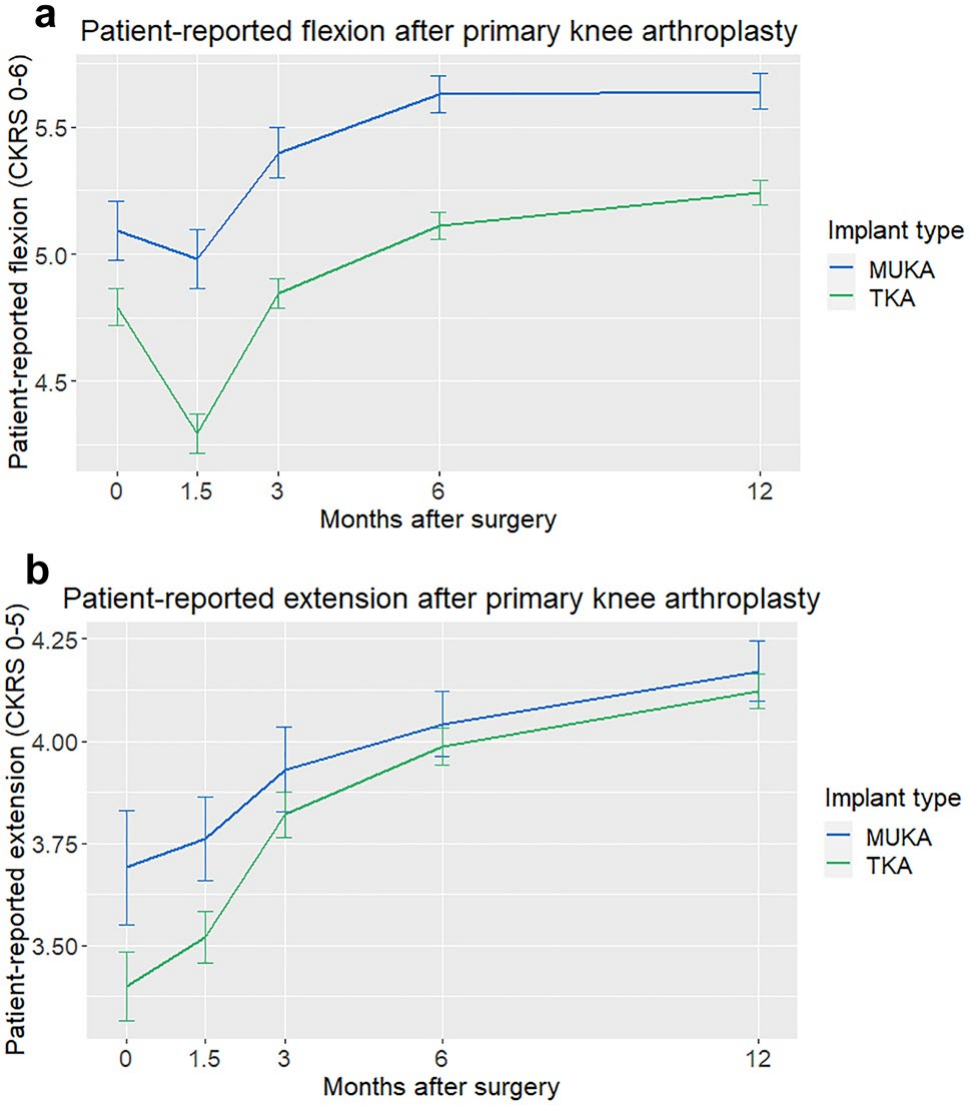
**a**, **b** Patient-reported **a** flexion and **b** extension after primary knee arthroplasty in the total sample, grouped by implant type (MUKA or TKA only), assessed with Copenhagen Knee ROM Scale. Whiskers denote mean ± 2 × std. error of the mean. Based on validation studies, flexion “4” corresponds to mean 101°, “5” to 121°, and 6 to 131°. In extension, “3” refers to mean 7°, “4” to 5°, and “5” to 1°

Patient satisfaction and willingness to repeat surgery were no different between hospitals at 1 year (Table 4). Aalborg patients gained more in general health (EQ-VAS change, *p* < 0.001). Aarhus patients had better knee extension at 1 year, but when adjustments for baseline motion were made, 1-year extension (and flexion) were independent of hospital. In contrast, MUKA was associated with a greater increase in 1-year flexion (+ 0.34 CKRS flexion points, *p* < 0.001) after baseline adjustments when compared to TKA, but not to increased extension (*p* = 0.3) (Fig. 3a, b).

### Hospital variation in results for comparable patients

**Fig. 4:**
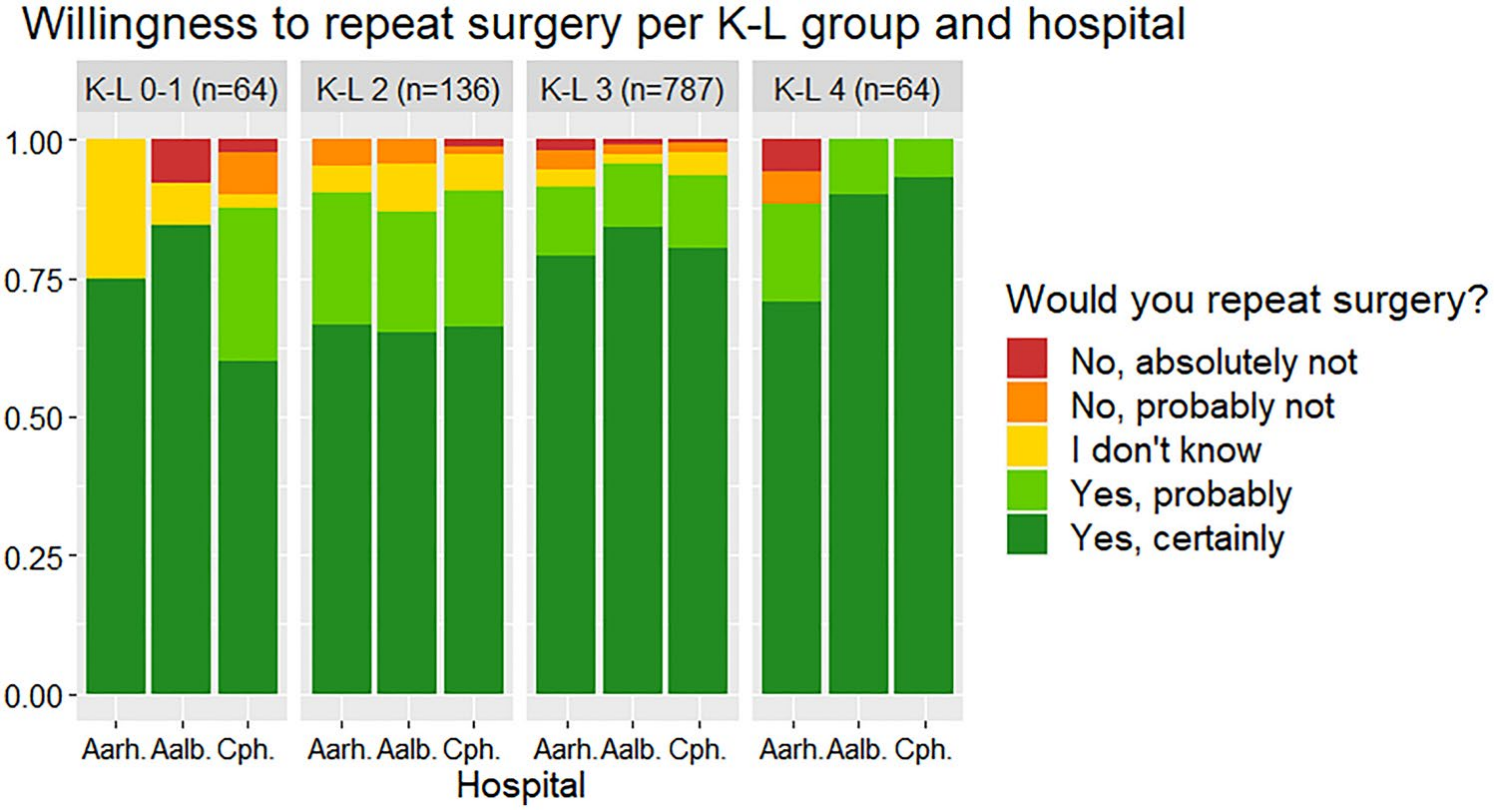
Willingness to repeat surgery at 1 year postoperatively grouped by Kellgren–Lawrence classification of preoperative knee OA and hospital, displayed as proportions

**Fig. 5:**
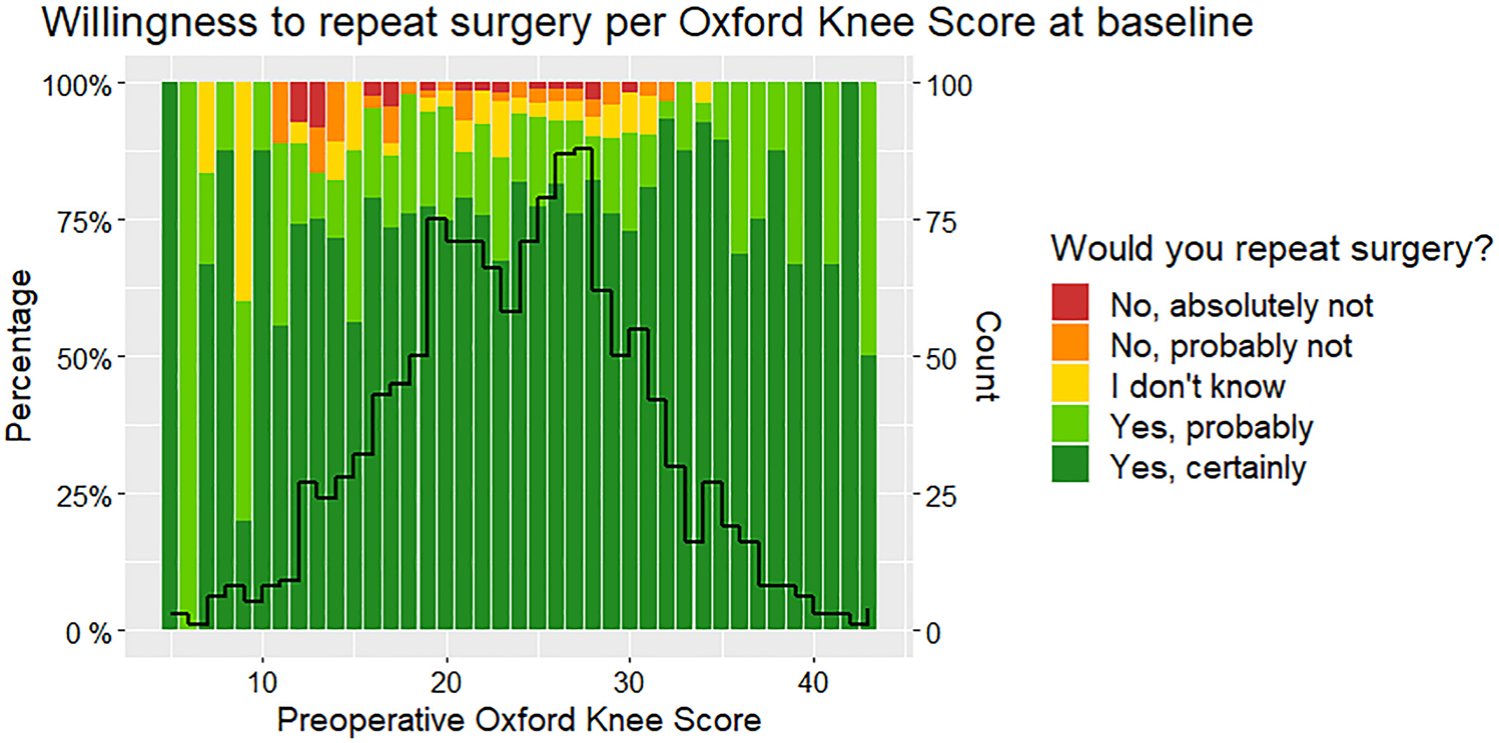
Willingness to repeat surgery at 1 year postoperatively per baseline Oxford Knee Score displayed as proportions of patients (total sample). Overlaying histogram depicts the number of patients with the specific preoperative OKS

When all patients were grouped by preoperative Ahlbäck or K–L classification, neither “willingness to repeat surgery”, 1-year OKS or “last postoperative OKS” varied significantly between hospitals (*p* = 0.09–1) (Fig. 4). An exception, however, was the “K–L 4” group of 64 patients, where the 17 Aarhus patients had 4–6 points lower 1-year OKS (CI 0.04–11) and 4–6 points lower “last postoperative OKS” (CI 0.03–10). With the frequent use of MUKA in Aarhus, it should be emphasized that only two “K-L 4” patients in Aarhus had MUKA and their 1-year OKS were 37 and 40, respectively. When patients were grouped by OKS at baseline (0–20, 21–30 and 30–48), none of the three aforementioned outcomes varied among hospitals (*P* = 0.2–0.5) (total sample displayed in Fig. 5). For patients with equivalent OKS results at 1 year (grouped by 10-point intervals), willingness to repeat surgery was independent of hospital (*p* = 0.2–0.8).

### Revision rate development

During the study period, the Danish Knee Arthroplasty Register observed a reduction in 2-year CRR variation between hospitals and regions (Table 6) [28].

**Table 6:** 2-Year cumulative revision rates in study hospitals and according regions

Hospital (region)	2-year CRR (%)			
	Pre-study period	Study period		
	Mean 2011–13	2016	2017	2018
Aarhus (Central Denmark Region)	1.9 (2.5)	3.2 (2.2)	**4.5** (2.0)	3.0 (1.9)
Aalborg (North Denmark Region)	1.6 (1.5)	2.4 (2.6)	2.9 **(3.4)**	1.7 **(3.9)**
Copenhagen (Capital Region)	**5.6 (4.7)**	**3.3 (2.8)**	3.1 (3.0)	**3.8 (3.9)**

Figures from the Danish Knee Arthroplasty Register. Bold figures denote the highest cumulative revision rate (CRR) of each year

## Discussion

Patients who underwent primary KA surgery across three Danish high-volume centres with a history of varied CRRs had comparable postoperative results when measured with PROMs, patient-reported knee ROM, patient satisfaction and willingness to repeat surgery. Across the three hospitals, patients with comparable preoperative radiographic knee OA or symptoms (OKS) had similar postoperative OKS results and were equally willing to repeat surgery, suggesting an overall homogeneity in the quality of treatment. This contradicts the conclusion that could be drawn from implant survival statistics alone, where high hospital CRRs are generally seen as indicative of inferior surgical outcomes. The CRRs provided by national KA registries are efficient means to detect poor performance of implants, techniques, hospitals or even surgeons, but they offer little information about treatment results in the far majority of patients; those who are not revised [8, 19, 22]. Outcome of surgery is not a yes-or-no question, but rather a wide spectrum ranging from a satisfied patient with a perfectly functioning prosthesis to an ill, infected patient in definite need of revision surgery. To quantify and eventually improve surgical quality, outcome evaluation should reflect this fact.

### Strengths and limitations

An observational cohort study was considered the most suitable design to explore the clinical reality behind the wide variations in Danish regional KA revision rates. With this design, however, no conclusions can be made regarding casual relationships. The three hospitals were selected to represent their respective regions; nonetheless, the results may not necessarily reflect the regional context. Despite the intention to invite virtually all primary KA patients, the average rate of participation was roughly 56%. Participants closely matched the demography and implant distribution of the total surgical population, but socioeconomic information was missing, general health data adhered from patient reports alone (EQ-5D-5L, smoking, alcohol consumption, height and weight), and patients who were unable to respond electronically were only allowed to participate in one-third of the study period [16, 7, 9]. In Aalborg, the larger gain in EQ variables was unexplained, but inclusion bias cannot be ruled out, given that only 38% participated here.

The study was strengthened by high response rates; 89% replied prior to surgery, and 97% of those participants responded after surgery (90% at 1 year).

The three hospitals differed in implant selection, introducing an important confounder which was inseparable from the hospital factor. In the total sample, there were variations in outcomes between MUKA and TKA patients, but although Aarhus used unicompartmental implants twice as frequently as the other two hospitals, implant-related differences were not readily apparent in the overall comparisons [13]. As an exception, Aarhus showed a tendency to have superior 1-year ROM. After adjusting for baseline flexion, the greater 1-year flexion gain in MUKA patients overall was + 0.3 CKRS points compared to TKA patients, which corresponds to about 5°, yet, the clinical relevance of differences in patient-reported ROM were not quantified as part of the CKRS scale validation [14].

In one low-revision hospital (Aarhus), there was a tendency of lower OKS change scores and fewer patients reaching MIC. Note, that the study cannot answer whether particular patients with poor progress would have benefited from revision surgery. The slightly higher OKS (+ 1.9) in Copenhagen patients at 6 weeks postoperatively may indicate a faster recovery that could be related to more frequent use of physiotherapy in rehabilitation. No differences were observed in other parameters, such as ROM, and when data were stratified by implant type, a different pattern was observed (Fig. 2b), suggesting that the finding may represent a sporadic and clinically insignificant variation [4].

Even though the SPARK study was motivated by regional differences in revision rates, revision surgery was not the main objective of the study, and the relatively few SPARK participants who underwent revision surgery during the first year were not expected to be representative of recent years’ practice. Yet, the contributions of the 28 patients who underwent revision were not disregarded, and efforts were made to compensate for the absence of 1-year PROMs in this group by use of transparent imputations.

The historical hospital differences in CRR were not confirmed in this cohort. This was anticipated given the sample size, but it is noteworthy that during the study period, variations in CRR did decrease at both the hospital and regional levels in Denmark [28]. This may represent a random variation or a general tendency. It cannot be ruled out that awareness of the ongoing SPARK study may have altered revision thresholds and patterns. As surgeon staffs and procedures have remained largely the same between past years and the study period, it seems unlikely that the quality of primary KA has changed at uneven pace in the three hospitals.

Having conducted this necessary comparison of primary KA results across Danish hospitals and regions, the next logical step in the search for explanations for revision rate variations would be a nationwide investigation of both revision thresholds and the benefit to patients from revision surgery. Such studies followed by discussions about revision indications and techniques might serve the patients with poor results as much as the ongoing attempts to refine primary knee replacement surgery.

## Conclusions

Patient-reported results 1 year after primary knee arthroplasty were comparable across three high-volume centres whose revision rates had varied for a decade. It follows, that hospital variance in revision rates does not necessarily reflect differences in the overall quality of primary surgery. Further studies focusing specifically on revision procedures should determine whether patients across regions and hospitals are offered revision surgery on the same clinical grounds.

## Data Availability

Raw data is available upon request.
